# Hypoxia-inducible factor-1 alpha is involved in RIP-induced necroptosis caused by *in vitro* and *in vivo* ischemic brain injury

**DOI:** 10.1038/s41598-017-06088-0

**Published:** 2017-07-19

**Authors:** Xiao-Sa Yang, Tai-Long Yi, Sai Zhang, Zhong-Wei Xu, Ze-Qi Yu, Hong-Tao Sun, Cheng Yang, Yue Tu, Shi-Xiang Cheng

**Affiliations:** 1 Tianjin Key Laboratory of Neurotrauma Repair, Institute of Traumatic Brain Injury and Neuroscience, Center for Neurology and Neurosurgery of Affiliated Hospital of the Logistics University of Chinese People’s Armed Police Force (PAP), No. 220 ChengLin Road, HeDong District, Tianjin, 300162 China; 2 Central Laboratory of Logistics University of PAP, No. 1 Huizhi Huan Road, DongLi District, Tianjin, 300393 China

## Abstract

Necroptosis, a novel type of programmed cell death, is involved in stroke-induced ischemic brain injury. Although studies have sought to explore the mechanisms of necroptosis, its signaling pathway has not yet to be completely elucidated. Thus, we used oxygen-glucose deprivation (OGD) and middle cerebral artery occlusion (MCAO) models mimicking ischemic stroke (IS) conditions to investigate mechanisms of necroptosis. We found that OGD and MCAO induced cell death, local brain ischemia and neurological deficit, while zVAD-fmk (zVAD, an apoptotic inhibitor), GSK’872 (a receptor interacting protein kinase-3 (RIP3) inhibitor), and combined treatment alleviated cell death and ischemic brain injury. Moreover, OGD and MCAO upregulated protein expression of the triggers of necroptosis: receptor interacting protein kinase-1 (RIP1), RIP3 and mixed lineage kinase domain-like protein (MLKL). The upregulation of these proteins was inhibited by GSK’872, combination treatments and RIP3 siRNA but not zVAD treatment. Intriguingly, hypoxia-inducible factor-1 alpha (HIF-1α), an important transcriptional factor under hypoxic conditions, was upregulated by OGD and MCAO. Similar to their inhibitory effects on aforementioned proteins upregulation, GSK’872, combination treatments and RIP3 siRNA decreased HIF-1α protein level. These findings indicate that necroptosis contributes to ischemic brain injury induced by OGD and MCAO and implicate HIF-1α, RIP1, RIP3, and MLKL in necroptosis.

## Introduction

Ischemic stroke (IS) is the second leading cause of death worldwide, with an annually increasing incidence rate, and it poses an enormous threat to public health^1^. During IS, neuronal cell death resulting from metabolic disturbance, excitotoxicity, and inflammatory response is a crucial element that aggravates IS severity and influences patients’ outcomes^2^. Therefore, exploring mechanisms of post-IS neuronal cell death is critical for clinical treatment.

There are multiple types of cell death, of which apoptosis (an active process of programmed cell death to maintain homeostasis) and necrosis (a passive and accidental process caused by pathological damage) are most widely known^3^. Recent evidence indicates that necroptosis, a novel form of programmed cell death, plays an important role in the brain, contributing to the pathological processes of cerebral ischemia/reperfusion injury and traumatic brain injury. However, necroptotic effect can be eliminated by the receptor-interacting protein 1 (RIP1) inhibitor Necrostatin-1 (Nec-1)^4, 5^. It was previously shown that necroptosis is mediated by the assembly of RIP1 and RIP3, forming the necrosome complex^6^, which recruits several molecules to execute necroptosis. Of these recruited molecules, mixed lineage kinase domain-like protein (MLKL) is considered to be an essential downstream protein, and its interaction ﻿with necrosome leads to necroptosis activation in IS^7^.

Growing evidence suggests that hypoxia-inducible factor-1 alpha (HIF-1α) is a major regulator of cellular and systemic homeostatic response to hypoxia and plays an essential role in pathophysiology of ischemic disease. Under ischemic conditions, the accumulation of HIF-1α promotes the transcription of multiple pro-survival proteins involved in energy metabolism, angiogenesis and neurogenesis, which serve as neuroprotectants against ischemic brain injury^8^. However, HIF-1α is also involved in post-stroke inflammatory responses, apoptosis and blood-brain barrier integrity loss^9^. Thus, the exact effects of HIF-1α and its mechanisms of action are still controversial and no studies have examined whether there is a link between necroptosis and HIF-1α in IS.

Oxygen-glucose deprivation (OGD) and middle cerebral artery occlusion (MCAO) models are most frequently used for the experimental study of IS *in vitro* and *in vivo*. In OGD model, normal cells cultured with abundant glucose and oxygen are transferred into an environment without glucose and oxygen for hours, resulting in the cell ischemic injury; after that, the injured cells are put back to the normal growth environment with adequate glucose and oxygen for a certain period to mimic the reperfusion of IS^10^. Similarly, MCAO model is performed by occluding the unilateral cerebral middle artery of animals with a suture to induce local ischemic brain injury, then removing the suture to execute reperfusion^11^. Numerous studies have validated that OGD and MCAO models are feasible to mimic the pathogenesis of IS and provide the experimental basis for the mechanism study of IS^12, 13^.

For this study, we used mice hippocampus neuron HT-22 cell line and C57BL/6 mice to establish OGD and MCAO models, respectively. HT-22 cells were derived from mice hippocampus region, which ensured the homology of subjects. And previous studies had demonstrated that HT-22 cells were suitable to conduct OGD model and explore the mechanism of ischemic injury *in vitro* ^14, 15^. GSK’872, a specific RIP3 inhibitor, was used to elucidate necroptosis effects in experimental IS. The expression and phosphorylation of necroptosis-associated proteins were detected to illuminate the mechanism of necroptosis post OGD and MCAO. Moreover, RIP3 and HIF-1α siRNA transfections *in vitro* were performed and the levels of HIF-1α and necroptosis-associated proteins were examined to determine the specific role of HIF-1α and its relationship with necroptosis.

## Results

### An optimized model of OGD and reoxygenation induces *in vitro* ischemia and reperfusion injury in HT-22 cells

**Figure 1:**
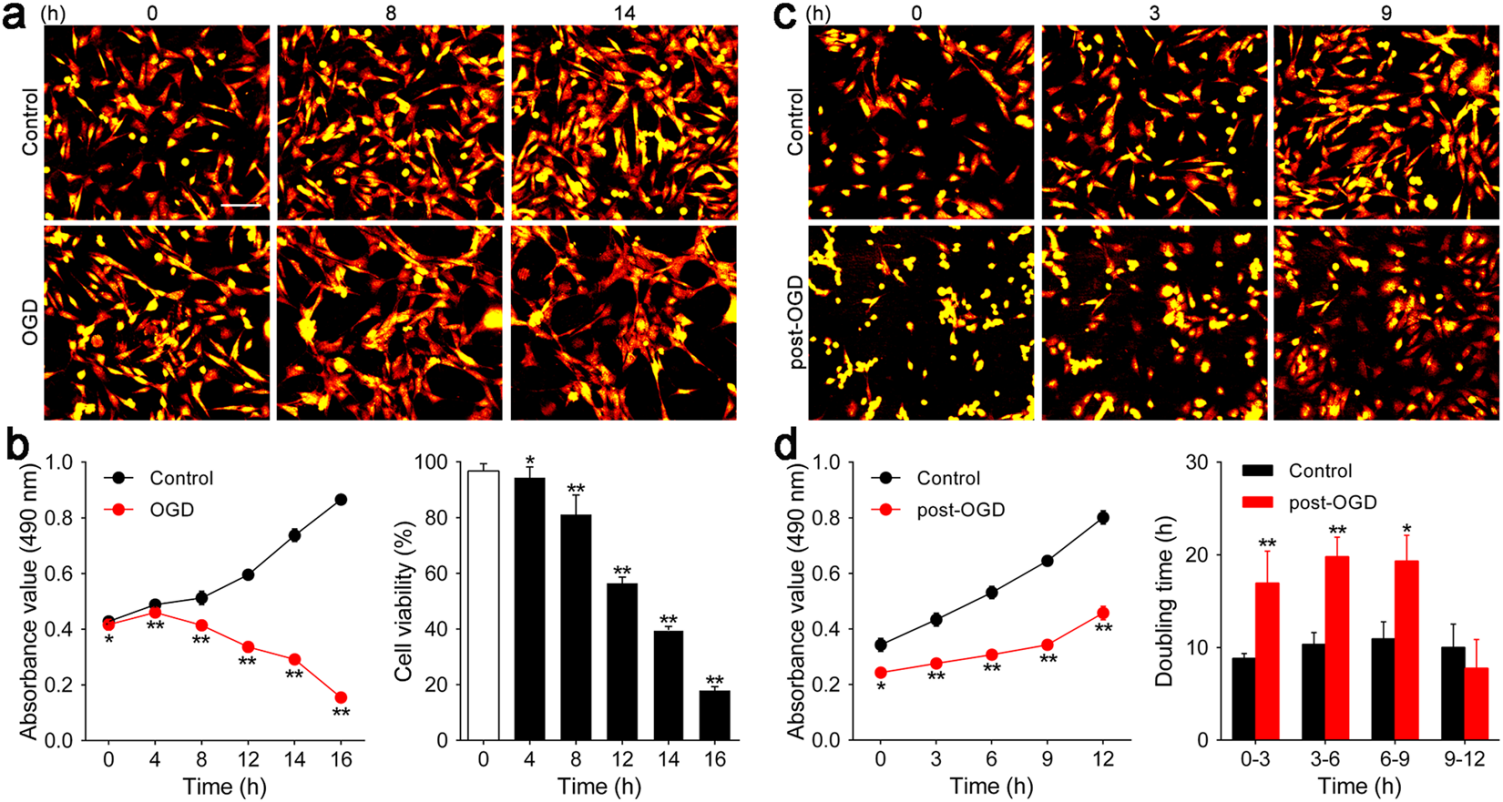
Determination of OGD and reoxygenation exposure times for HT-22 cells. (**a** and **c**) Digital hologram microscopy was used to acquire representative images showing changes in HT-22 cell number and morphology during the OGD and reoxygenation phases. Scale bar = 100 μm. (**b**) A growth curve was generated using absorbance values (490 nm) and cell viability was detected by MTS assay at 0, 4, 8, 12, and 16 h of OGD. (**d**) HT-22 cell absorbance values (490 nm) at 0, 3, 6, 9, and 12 h of reoxygenation were used to construct the growth curve. Doubling time during 0–3, 3–6, 6–9, and 9–12 h of reoxygenation was calculated. *p < 0.05, **p < 0.01 *vs*. control group. Also see Supplementary Figures S1, S2 and Videos S1, S2.

A growing body of literature suggests that oxygen and glucose deprivation within normal cells is a useful model of ischemia and reperfusion injury *in vitro* ^16^. Previous studies have established appropriate OGD and reoxygenation exposure times to produce successful models; however, we previously found that OGD and reoxygenation exposure times are variable for different type of cells potentially due to their dissimilar cell growth and differentiation characteristics^17, 18^. To confirm the most suitable OGD and reoxygenation exposure times for HT-22 cells, we used digital holographic microscopy, light microscopy and MTS assay to evaluate alterations in cell morphology and viability. In the OGD period, HT-22 cell morphologic changes, including flotation, elongation, irregularity and sparseness, occurred as OGD exposure time increased, whereas control group cells became more closely arranged with regular shapes and minimal flotation (Fig. 1a, Supplementary Figure S1 and Video S1). MTS assay showed the growth curves of control and OGD group cells were opposite in direction. Moreover, it revealed that the viability of cells exposed to 4, 8 and 12 h of OGD decreased to 94.36 ± 1.94% (*p* < 0.05), 81.11 ± 3.52% and 56.40 ± 1.13% (both *p* < 0.01), respectively, whereas cells exposed to 14 and 16 h of OGD suffered dramatically with cell viabilities of 39.57 ± 0.68% and 17.86 ± 0.73% (both *p* < 0.01), respectively; however, 16 h of OGD resulted in excessive cell death (Fig. 1b), which was in agreement with morphologic alterations. Considering the above results, we determined the exposure duration of 14 h during the OGD phase in subsequent experiments.

During the reoxygenation stage, cells began to adhere to the plate and resume normal shapes but still grew slowly after 14 h of OGD exposure compared to control group cells. A significant increase in cell number was present only at 12 h of reoxygenation, whereas control group cells grew at an appropriate speed (Fig. 1c, Supplementary Figure S2 and Video S2). Similar results were obtained from MTS assay, which demonstrated that post-OGD cells grew more slowly with significantly lower absorbance than cells without OGD exposure (0 h: 0.24 ± 0.01 *vs*. 0.34 ± 0.01, 3 h: 0.28 ± 0.01 *vs*. 0.43 ± 0.01, 6 h: 0.31 ± 0.01 *vs*. 0.53 ± 0.01, 9 h: 0.34 ± 0.01 *vs*. 0.65 ± 0.01, 12 h: 0.46 ± 0.01 *vs*. 0.80 ± 0.01, all *p* < 0.01). Moreover, cell doubling time during 0–3 h, 3–6 h and 6–9 h post-OGD was much longer than that of control cells [0–3 h: (16.95 ± 1.72) *vs*. (8.84 ± 0.26) h, 3–6 h: (19.81 ± 1.05) *vs*. (10.33 ± 0.64) h, 6–9 h: (19.34 ± 1.38) *vs*. (10.94 ± 0.92) h, all *p* < 0.01]; however, cell doubling time during 9–12 h post-OGD showed a remarkable decrease and was not significantly different from control cells [(7.93 ± 1.48) *vs*. (10.02 ± 1.25) h, *p* > 0.05], indicating that cells at 9–12 h of reoxygenation had returned to normal growth (Fig. 1d). On the basis of these results, 9 h was chosen as the appropriate reoxygenation time.

### GSK’872, zVAD and combination treatment attenuate HT-22 cells death induced by OGD and reoxygenation

**Figure 2:**
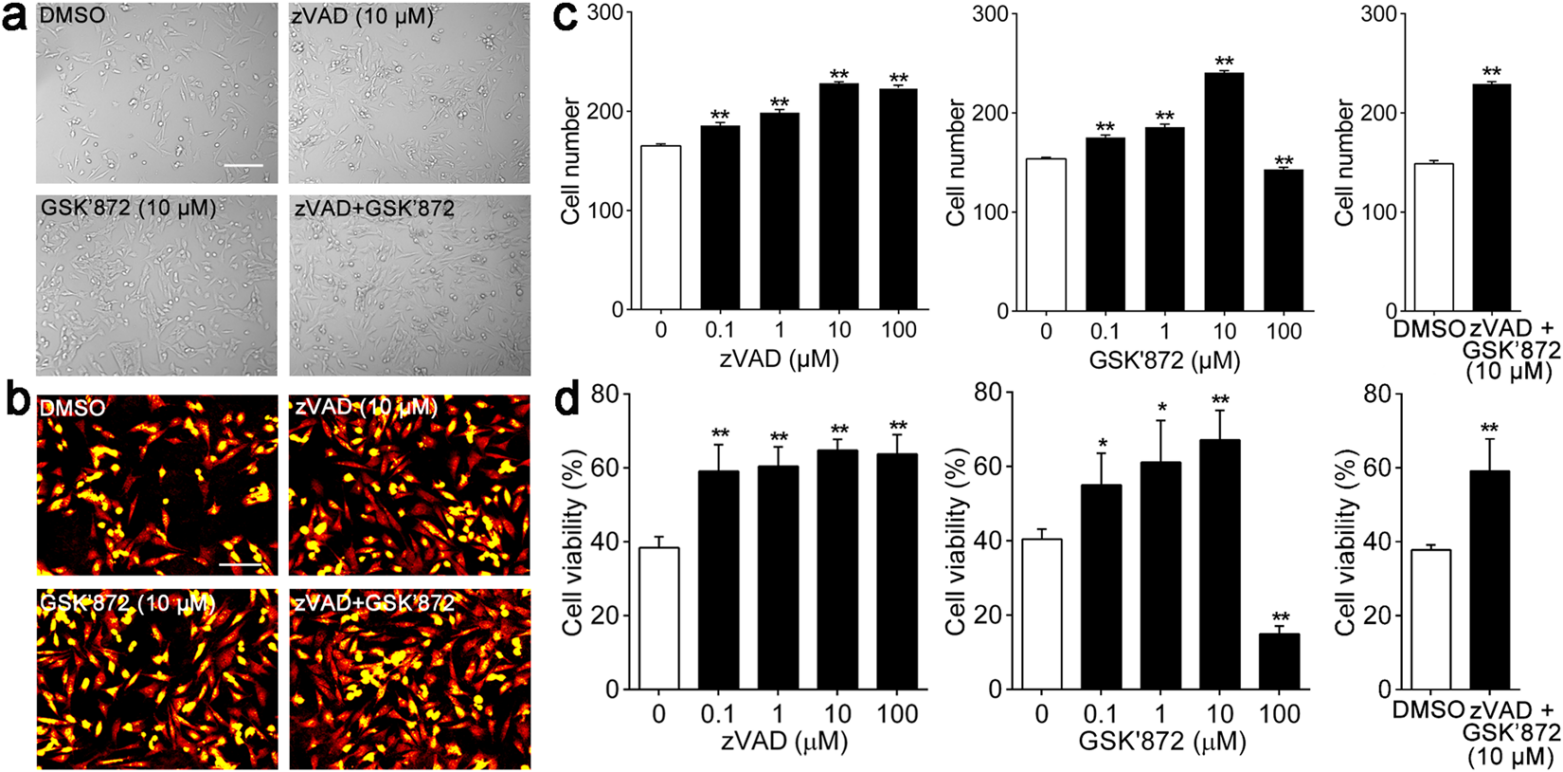
zVAD, GSK’872 and combinative intervention alleviated morphologic alterations and attenuated cell death induced by OGD and reoxygenation in HT-22 cells. (**a** and **b**) Representative images of HT-22 cells morphologic changes during reoxygenation were captured by light microscope and digital hologram microscope. Scale bars = 100 μm. (**c**) Cell viability was determined by MTS assay after 9 h of reoxygenation. (**d**) Alterations of HT-22 cell number during reoxygenation were quantified and analyzed by HoloStudio 2.6 software. *p < 0.05, **p < 0.01 *vs*. control group. Also see Supplementary Figure S3 and Video S3.

A previous study demonstrated that the OGD and reoxygenation model mimicking hypoxic-ischemic injury *in vitro* induces multiple types of cell death, including apoptosis and necroptosis^19^. To investigate which type of cell death was evoked by the OGD and reoxygenation model in HT-22 cells, zVAD (an apoptosis inhibitor) and GSK’872 (a necroptosis inhibitor) were utilized. After 14 h of OGD, cells were treated with different concentrations of zVAD, GSK’872 and a combination of zVAD and GSK’872 during 9 h of reoxygenation. As shown in Fig. 2a and b, cells treated with zVAD, GSK’872 and a combination of zVAD and GSK’872 (except for cells treated with GSK’872 100 μM alone) returned to normal morphology unlike cells without treatment (Supplementary Figure S3 and Video S3). Moreover, cell numbers increased significantly with zVAD (0.1, 1, 10 and 100 μM), GSK’872(0.1, 1, 10 μM) and combination treatment (Fig. 2c). MTS assay results were in agreement with changes in cell number and morphology. Viability of HT-22 cells treated with zVAD (0.1, 1, 10 and 100 μM) and GSK’872 (0.1, 1 and 10 μM) was significantly increased compared with that of control cells. Cells treated with 10 μM zVAD or 10 μM GSK’872 had the opposite effect [zVAD: 0.1 μM (59.08 ± 3.61%), 1 μM (60.44 ± 2.66%), 10 μM (64.79 ± 0.48%), 100 μM (63.75 ± 2.64%) *vs*. control (38.43 ± 1.49%), all *p* < 0.01; GSK’872: 0.1 μM (55.02 ± 4.29%), 1 μM (61.18 ± 5.60%), 10 μM (67.17 ± 3.99%), 100 μM (14.94 ± 1.06%) *vs*. control (40.43 ± 1.38%), *p* < 0.05 or <0.01]. Thus, we selected 10 μM as the concentration of each reagent in the combination treatment in subsequent experiments. Notably, combination treatment increased cell viability in comparison to that of control cells (59.14 ± 4.35 *vs*. 37.82 ± 0.68, *p* < 0.01; Fig. 2d).

### Necroptosis is critical for brain damage in mouse ischemic stroke

**Figure 3:**
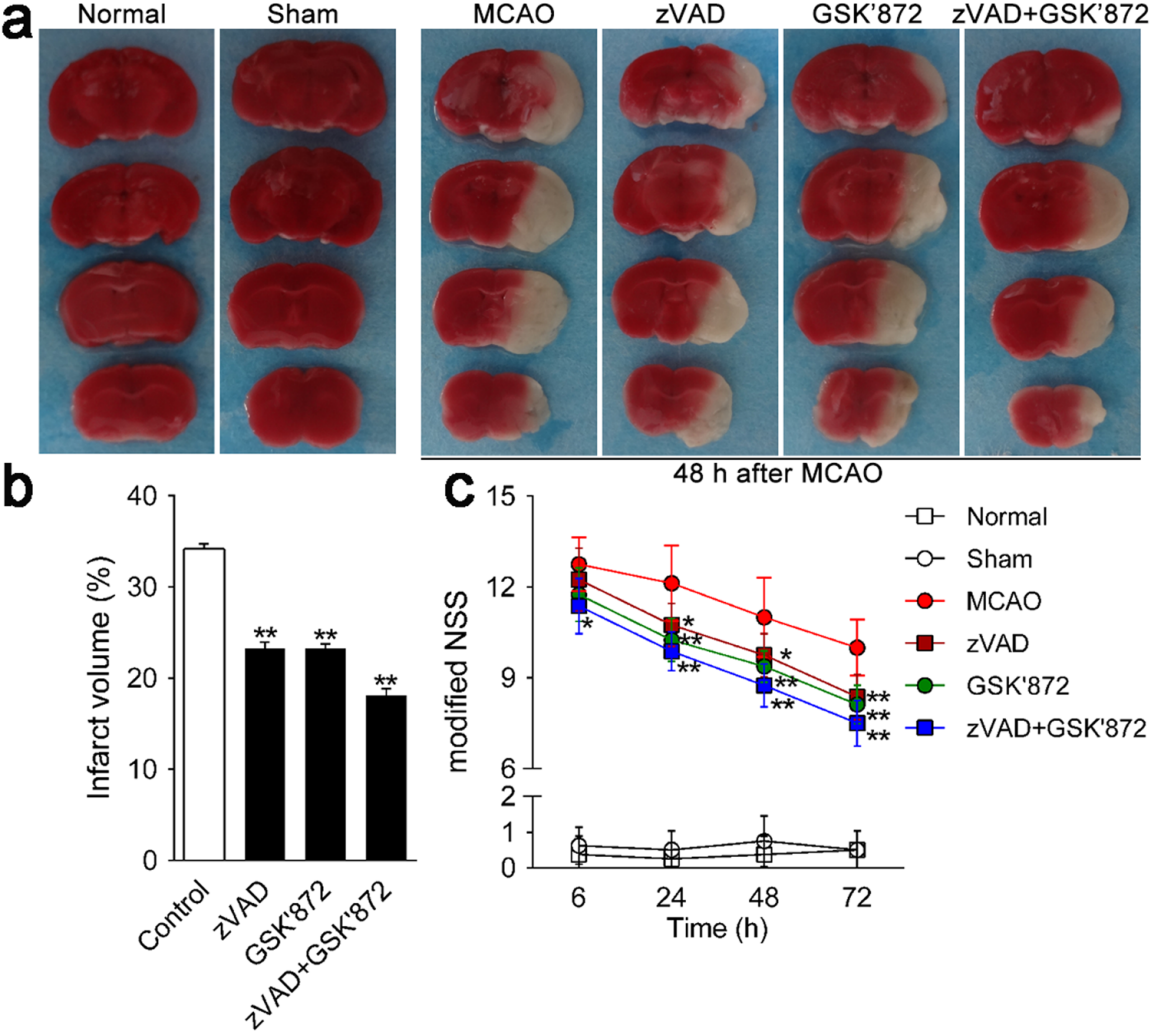
GSK’872 and combinative intervention attenuated ischemic brain injury and neurological deficits induced by MCAO in mice. (**a**) Ischemic areas were visualized by TTC staining at 48 h after MCAO (*n* = 3 per group). Normal tissue stained rose-pink, whereas infarct area appeared white. (**b**) Infarct volume was quantified by Image-Pro plus 6.0 software. (**c**) Neurological deficits were evaluated by mNSS at 6, 24, 48 and 72 h after MCAO (*n* = 9 per group). Data are presented as the mean ± SD. *p < 0.05, **p < 0.01 *vs*. control group.

RIP3 commonly executes neuronal necroptotic death in stroke^20^. To determine whether inhibition of RIP3 activity by GSK’872 ameliorates ischemic stroke damage *in vivo*, we used an MCAO model and detected the resulting brain injury by standard TTC staining, in which infarct area appeared white, whereas viable non-infarcted tissue stained rose-pink (Fig. 3a). As shown in Fig. 3b, the percentage of the infarct volume in GSK’872-treated mice (23.29 ± 0.29%) was significantly smaller than that of non-treated mice (34.18 ± 0.33%) at 48 h after MCAO (*p* < 0.01). We found a similar reduction of infarct volume in zVAD-treated mice (23.18 ± 0.38%) compared with that of non-treated mice (*p* < 0.01). Interestingly, the combination treatment also markedly reduced the infarct volume (18.16 ± 0.41%) compared with that of non-treated mice (*p* < 0.01). Furthermore, differences in brain damage were functionally relevant with reduced infarct volume correlating with lower mNSS scores (Fig. 3c).

### Intervention with GSK’872 and combination treatment inhibit the relative expression of necroptosis-associated proteins *in vitro* and *in vivo*

**Figure 4:**
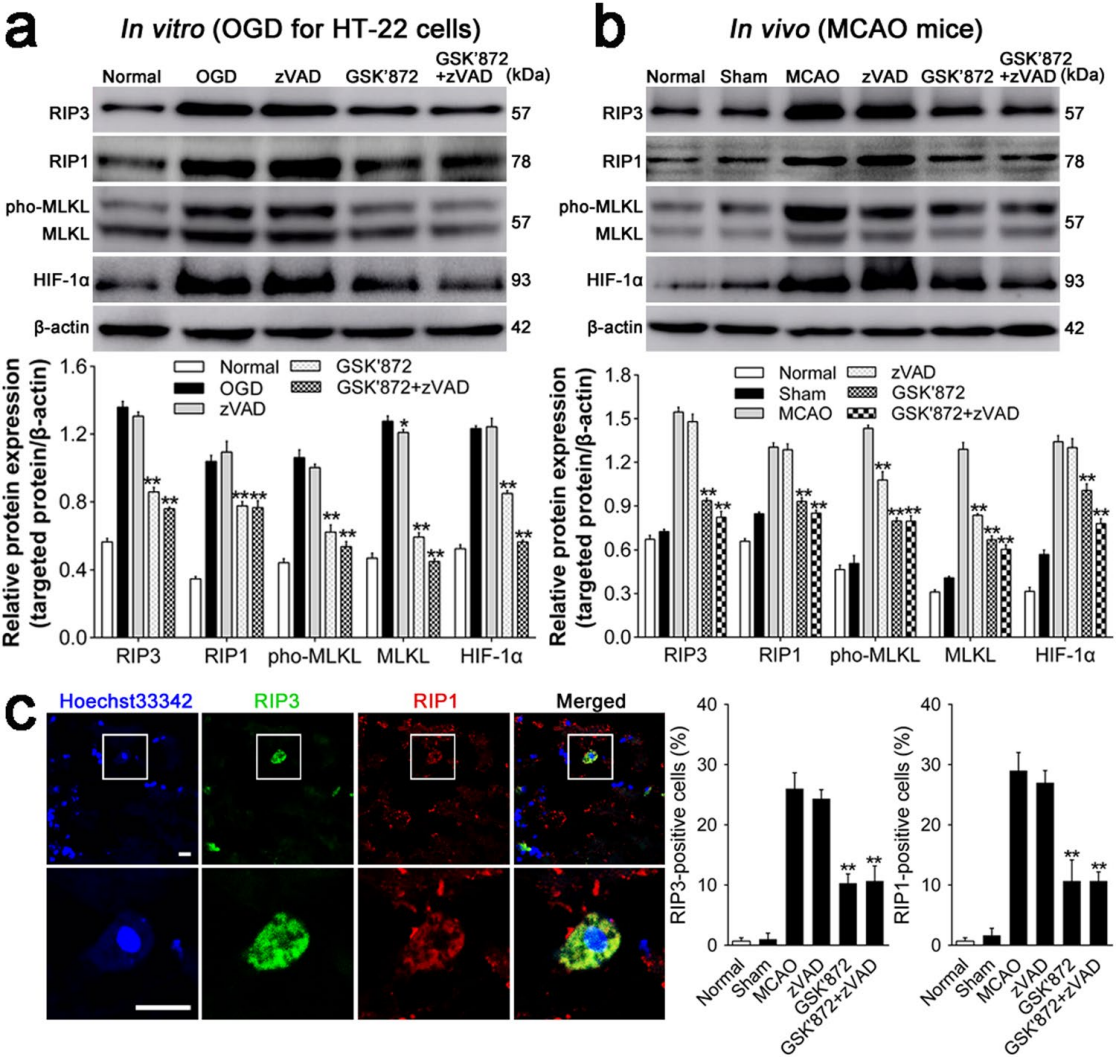
GSK’872 and combinative intervention downregulated HIF-1α and necroptosis-associated proteins. (**a** and **b**) Relative protein expression *in vitro* and *in vivo* was detected by Western Blotting assay, measured as the ratio to *β*-actin, and analyzed using Scion Image 4.0 software. (**c**) (Left) Representative RIP3 (green) and RIP1 (red) staining co-localized in the plasma of the infarct region of mice. Nuclei were visualized with Hoechest33342. Scale bar = 10 μm. (Right) Quantitative analysis of RIP3- or RIP1-positive cells. Data are reported as the mean ± SD. *p < 0.05, **p < 0.01 *vs*. OGD or MCAO group.

RIP1, RIP3 and MLKL are thought to be involved in the formation and activation of necrosome, which plays a crucial role in the necroptotic signaling pathway^21^. Moreover, the phosphorylation of MLKL is confirmed to be indispensable to the activation of necrosome^7^. Thus, we explored the relative expression of RIP1, RIP3, MLKL and the phosphorylated MLKL *in vitro* and *in vivo* subjected to different interventions. Western blotting assay indicated that GSK’872 treatment decreased RIP1, RIP3 and MLKL expression and inhibited the phosphorylation of MLKL compared with the non-treated group *in vitro* and *in vivo* (all *p* < 0.01). Similar downregulation occurred under combination treatment in comparison with the non-treated group (all *p* < 0.01). RIP1, RIP3 and the phosphorylated MLKL showed no obvious change when treated with zVAD compared with the non-treated group (Fig. 4a and b); however, MLKL was downregulated with zVAD treatment *in vitro* (*p* < 0.05) and *in vivo* (*p* < 0.01). Moreover, fluorescence images demonstrated that RIP3 (green) and RIP1 (red) were co-located in the cytoplasm within the infarct region post-stroke. The percentages of RIP1- and RIP3-positive cells were increased significantly post-MCAO and exhibited a notable reduction with GSK’872 and combination treatment compared with the non-treated group (all *p* < 0.01). Comparison between zVAD treatment and non-treated groups revealed no significant difference (Fig. 4c).

### Downregulation of HIF-1α is induced by GSK’872 and combination treatment after ischemia injury *in vitro* and *in vivo*

Previous studies have demonstrated that HIF-1α and its downstream proteins are involved in hemorrhagic transformation following transient MCAO and suppression of HIF-1α attenuates brain damage and neurological deficit^22^. Therefore, we assessed the relative expression of HIF-1α when treated with different reagents following OGD and MCAO. As shown in Fig. 4a and b, ischemia damage caused by OGD and MCAO dramatically upregulated HIF-1α expression. Surprisingly, GSK’872 treatment significantly decreased HIF-1α expression compared with no treatment. Moreover, HIF-1α downregulation was more pronounced with combination treatment *in vitro* and *in vivo* (both *p* < 0.01). There was no significant difference in HIF-1α level between the zVAD-treated and non-treated groups.

### RIP3 siRNA decreases the protein level of HIF-1α post ischemia injury *in vitro*

**Figure 5:**
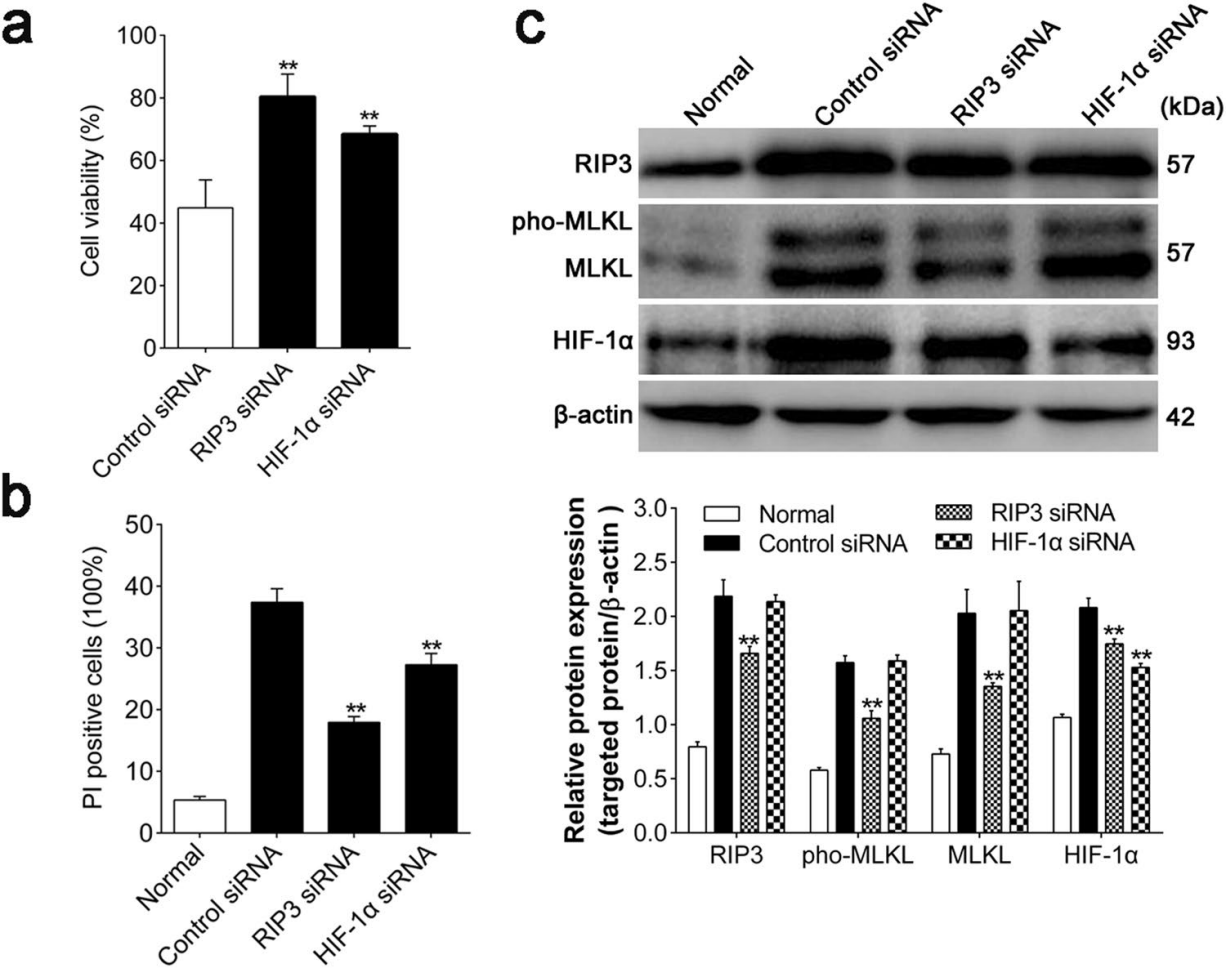
RIP3 siRNA alleviated cell death and decreased the protein level of HIF-1α post OGD in HT-22 cells. (**a**) The viability of transfected HT-22 cells was detected by MTT assay after 9 h of reoxygenation. (**b**) The alterations of cell death with different siRNA transfections were analyzed by flow cytometry. (**c**) The protein expressions of HIF-1α, RIP3, MLKL and phosphorylated MLKL were determined by Western Blotting assay, normalized to β-actin levels. Control siRNA represented nontargeting siRNA transfection and pho-MLKL was the abbreviation of the phosphorylated MLKL. **p < 0.01 *vs*. Control siRNA group. Also see Supplementary Figure S4.

In order to further explore the relationship between HIF-1α and necroptosis, we knocked down RIP3 and HIF-1α via siRNA transfection *in vitro* and analyzed the alterations of cell viability and cell death using MTT assay and flow cytometry, respectively. Besides, we also detected the expression of RIP3, MLKL, phosphorylated MLKL and HIF-1α in the absence of RIP3 and HIF-1α. MTT assay indicated the either RIP3 or HIF-1α siRNA increased the viability of OGD injured HT-22 cells when compared with nontargeting siRNA transfected cells (both *p* < 0.01; Fig. 5a). Consistent with the alteration of cell viability, flow cytometry showed that cell death was significantly attenuated when either RIP3 or HIF-1α was knocked down (both *p* < 0.01; Fig. 5b and Supplementary Figure S4). As shown in Fig. 5c, OGD obviously upregulated RIP3, MLKL, phosphorylated MLKL and HIF-1α while RIP3 siRNA decreased the levels of those proteins significantly (all *p* < 0.01). However, except for the self-inhibition (*p* < 0.01), HIF-1α siRNA didn’t affect the expression of RIP3, MLKL and phosphorylated MLKL in comparison to nontargeting siRNA.

## Discussion

In this study, we investigated the optimal hypoxic-ischemic condition *in vitro*. We found that viability of HT-22 cells exposed to 14 h of OGD decreased to 39.25 ± 0.63%. This decreased cell viability was accompanied by hypoxia-induced morphologic changes. Moreover, the turning point of cell recovery during reoxygenation was 9 h. Improvements in cell viability and morphology showed cells grew slowly before 9 h but recovered obviously during 9–12 h of reoxygenation. Thus, we determined that an OGD exposure time of 14 h and a reoxygenation exposure time of 9 h would allow treatment effects to be recognized without interference from the naturally rapid cell growth of HT-22 cells.

IS-induced ischemic brain injury is involved in multiple molecular and biological mechanisms including necroptosis, a newly discovered type of programmed cell death that has not yet to be fully elucidated^23^. In this study, we used MCAO and OGD models for experimental IS. With the administration of zVAD, GSK’872, and combination treatment and RIP3 siRNA transfection, we confirmed the existence of necroptosis after transient focal cerebral ischemia and deprivation of oxygen and glucose and demonstrated necroptosis mechanisms via alterations in RIP1, RIP3, MLKL and phosphorylated MLKL protein expression. Additionally, we conducted a preliminary exploration of the relationship between HIF-1α and necroptosis by RIP3 and HIF-1α siRNA transfection *in vitro*.

Necroptosis and apoptosis are elementary neuronal cell death mechanisms that operate within the ischemic zone post-IS^24^. A previous study showed that Nec-1 and Gly^14^-humanin (HNG, an apoptosis inhibitor) reduced infarct volume and ameliorated neurological deficits induced by MCAO and demonstrated that combined treatment had more neuroprotective effects^5^. These findings suggest that necroptosis as well as apoptosis were involved in ischemic brain injury. Moreover, Nec-1 pretreatment inhibited necroptotic cell death induced by OGD and decreased the production of pro-inflammatory cytokines to oppose ischemic injury, which suggests that necroptosis was involved in ischemic insults post-IS^25^. In this study, we observed that cell death, local brain infarction, and neurological deficits induced by MCAO and OGD were attenuated dramatically when treated with zVAD (an apoptosis inhibitor) or GSK’872 (an RIP3 inhibitor). The combined zVAD/GSK’872 treatment showed stronger effects than either single inhibitor treatment. Moreover, RIP3 siRNA significantly ameliorated the cell injury and attenuated cell death as with the above inhibitors. This suggests that necroptosis and apoptosis were related to neuronal cell death post-IS and necroptotic and apoptotic inhibitors may act synergistically to promote neuroprotection against ischemic brain injury caused by MCAO and OGD.

Multiple intercellular pathways regulate necroptosis, which is characterized by mitochondria dysfunction, cell swelling, cell membrane disruption, and the release of cytoplasmic contents^26, 27^. In a previous study, neuronal necroptotic cell death was induced by OGD, which also elevated RIP1 and RIP3 expression and RIP1-RIP3-MLKL interaction^21^. However, Nec-1, RIP3-, or MLKL-siRNA inhibited RIP1-RIP3-MLKL interaction, dampened necroptosis and downregulated the expression of RIP3 and MLKL. Moreover, another study has shown that following phosphorylation and tetramerization by the RIP1 and RIP3 assembly, MLKL is translated to lipids in the cell membrane causing sodium influx, cell swelling, and cell membrane breakage, which results in necroptosis^28^. Our study showed that RIP1, RIP3, MLKL and the phosphorylated MLKL expression was upregulated by ischemic injury *in vitro* and *in vivo* and demonstrated that GSK’872 and RIP3 siRNA induced the downregulation of these proteins and alleviated ischemic insults. Our findings provide evidence for RIP1, RIP3 and MLKL involvement in post-IS necroptosis and demonstrate that GSK’872 as well as RIP3 siRNA promoted neuroprotection via inhibiting RIP1, RIP3 and MLKL and suppressed the phosphorylation of MLKL similar to previously studied inhibitors.

HIF-1α is an important transcriptional factor that is stabilized by intercellular hypoxic environments. HIF-1α regulates the transcription of more than 100 downstream hypoxia and ischemia genes^29^ and participates in complicated post-IS ischemic brain injury processes, including oxidative stress, cell death, adaptive response and regeneration^30^. However, whether HIF-1α plays a neuroprotective role or not is still controversial. Some studies show that HIF-1α inhibition reversed the beneficial effects of valproate^31^. Other studies indicate that edaravone ameliorated hippocampal CA1 injury and neural stem/progenitor cells death by inhibiting ROS generation and HIF-1α expression. ROS generation has been shown to play a vital role in the activation of HIF-1α^32^, suggesting that ROS-dependent HIF-1α activation results in cell death after cerebral ischemia^33^. Moreover, the suppression of HIF-1α by baicalein is mediated by inhibiting ROS generation and PI3-kinase/Akt activation, which suggests that ROS acts as an upstream regulator of HIF-1α accumulation and stabilization^34^. In the present study, we observed ischemic injury improvement and RIP1, RIP3, MLKL and phosphorylated MLKL reduction following GSK’872 treatment and RIP3 siRNA transfection, which coincided with the downregulation of HIF-1α. Moreover, HIF-1α siRNA attenuated the ischemic injury but didn’t affect the protein expression of RIP3, MLKL and phosphorylated MLKL *in vitro*, which suggests that, on one hand, the accumulation of HIF-1α in hypoxic conditions had an adverse effect on *in vitro* and *in vivo* IS pathological process in agreement with previous reports. On the other hand, HIF-1α may be the downstream effector and indirectly implicated in necroptosis which deteriorated ischemic brain injury. Considering previous studies and our data, we propose that the relationship between HIF-1α and necroptosis may be facilitated by ROS, which participates in the activation of HIF-1α and regulation of necroptosis.

Little is known about HIF-1α molecular mechanisms involved in necroptotic signaling in IS, but recent reports indicate that alterations of mitochondrial function and ROS generation are mediators of RIP kinases-dependent necroptosis^35, 36^. Huang CY *et al*. documented that RIP-dependent necroptosis induced by hypoxia was eliminated by glucose and pyruvate, which prevented mitochondrial ROS generation and attenuated RIP1 and RIP3 interaction. These glucose and pyruvate effects ultimately led to necroptosis resistance and morphological recovery by hypoxic-damaged cells, indicating that ROS generation contributed to necroptotic process induced by hypoxia^37^. Moreover, Yuan L *et al*. demonstrated that OGD treatment induced necroptotic cell death, increased levels of ROS and neuronal oxidative stress-associated histone deacetylase 6, and histone deacetylase 6 inhibitor diminished OGD-induced necroptosis by inhibiting ROS generation^38^. Those results suggest that ROS may play a crucial role in necroptosis regulation.

Our data show that necroptosis relates to the ischemic injury induced by OGD and MCAO by upregulating RIP1, RIP3, MLKL and phosphorylated MLKL. We also found that GSK’872 and RIP3 siRNA blocks RIP1-RIP3-MLKL-dependent necroptosis and promotes neuroprotective effect following OGD and MCAO. Moreover, we observe that HIF-1α exerts adverse effects relevant to ischemic brain injury *in vitro* and *in vivo*. Interestingly, the simultaneous alterations of HIF-1α and necroptosis-associated proteins suggest, for the first time, that HIF-1α is involved in necroptosis. These findings provide a novel perspective for studies of IS necroptosis mechanisms and identify necroptosis as a potential target for IS clinical therapy.

## Materials and Methods

### Cell culture

Murine hippocampal neuronal HT-22 cells were obtained from HuiYing Biological Technology (Shanghai, China) and cultured with Dulbecco’s modified essential medium (DMEM, BasalMedia, Shanghai, China) containing 10% fetal bovine serum (FBS, TianHang biotech, Huzhou, China) in a humidified atmosphere of 5% CO_2_ at 37 °C. The medium was refreshed every 2 days, and cell passages were performed at 90% confluence. The cells were cultured conventionally for 7 days before experiments were performed.

### OGD/reoxygenation model

*In vitro* ischemia was induced in HT-22 cells by OGD as previously described with some optimization^39^. We chose HT-22 cells for the OGD model owing to their origin from the hippocampus in mice and their widespread use in experimental models of the brain^40^. Briefly, cells were washed twice with DMEM without glucose (BasalMedia, Shanghai, China), transferred to a DMEM medium without glucose and cultured in a hypoxic atmosphere of 95% N_2_ and 5% CO_2_ for a group-assigned exposure period. After the OGD procedure, cells were returned to a normoxic incubator with DMEM medium to stimulate reoxygenation. In the OGD phase, cells were randomized to five groups in accordance with exposure time of 4, 8, 12, 14 and 16 h. For the reoxygenation phase, cells were assigned to four groups with reoxygenation time of 3, 6, 9 and 12 h. Cells in control group were maintained in DMEM for the same time as cells during the OGD and reoxygenation phases. Following the OGD and reoxygenation procedures, cells were collected for subsequent analysis.

### Drug administration

zVAD-fmk (zVAD, Selleck, Houston, USA) and GSK’872 (Merck Millipore, Darmstadt, Germany) were dissolved in DMSO (<0.1%) and diluted in DMEM at four concentrations (0.1, 1, 10 and 100 μM). To test the reagent effects, cells were treated with zVAD (0.1, 1, 10 and 100 μM), GSK’872 (0.1, 1, 10 and 100 μM), or a combination of zVAD and GSK’872 at the beginning of reoxygenation. Moreover, cells were divided into subgroups according to zVAD and GSK’872 concentrations. The concentrations of each reagent in the combination treatment were determined by subsequent assays. Cells in control group were treated with DMSO only during the indicated period of reoxygenation.

### Cell viability assay

Cell viability was assessed with the MTS [3-(4,5-dimethylthiazol-2-yl)-5-(3-carboxymethoxyphenyl)-2-(4-sulfophenyl)2-H-tetrazolium] assay as described previously^5^. Cells were seeded into 96-well plate at a density of 3,000/well. Following the OGD and reoxygenation, 20 μl of MTS (5 mg/ml) per well was added, and cells were incubated for additional 2 h. Then, the plates were placed in a microplate autoreader (Thermo, NY, USA), and the absorbance at a wavelength of 490 nm was measured. Cells were seeded and assayed in triplicate for each experimental condition. Cell viability was calculated as the ratio of absorbance in treated cells to that in control cells. Cell growth curves were obtained using GraphPad Prism 6.0.

### Digital holographic microscopy

Cells were seeded into 6-well plates at a density of 1 × 10^5^/ml and cultured for 24 h before OGD/reoxygenation or sham procedures. At the onset of reoxygenation, the medium was replaced with DMEM containing the above-mentioned reagents, and the cells were transferred to a digital holographic microscope (HoloMonitor^TM^ M4, Phase Holographic Imaging AB, Lund, Sweden) positioned in an incubator at 37 °C in 5% CO_2_. The digital holographic microscope recorded 3D cell structure through the interfering wave fronts induced by the exposure to a 0.8 mW HeNe laser (633 nm). For each experimental group, an imaging area was selected randomly, and time-lapse images were captured at 20-min intervals. HoloStudio 2.6 was used to analyze cell number alterations during imaging periods.

### Transient transfections of RIP3 and HIF-1α siRNA

HT-22 cells were cultured under normal growth condition for 24 h prior to transfection. SiRNAs targeting mouse RIP3 (AuGCT, Beijing, China) and HIF-1α (AuGCT, Beijing, China) were synthesized with the following sequences: 5′-TGG CAC TCC TCA GAT TCC ACA TAC T-3′ and 5′-AAG CAU UUC UCU CAU UUC CUC AUG G-3′, respectively. Nontargeting siRNA with the sequence 5′-UUC UCC GAA CGU GUC ACG U-3′ was used as blank control. After incubated with Lipofectamine^®^ 2000 (Invitrogen, CA, USA) in Opti-MEM (BasalMedia, Shanghai, China) for 20 minutes at room temperature, the siRNAs were transferred into HT-22 cells for 24 h in normoxic environment. Then the transfected cells were arranged to conduct OGD model.

### Flow cytometry

The transferred cells were collected at 9 h after OGD. After washed with PBS twice, cells were incubated with 100 μl PI (1:1000 dilution; Life Technologies, CA, USA) for 15 min at room temperature in the dark. After incubation, each sample was added with 400 μl 1× annexin binding buffer (Invitrogen, CA, USA) and immediately analyzed by flow cytometry (BD Biosciences, MD, USA).

### Animals and experimental groups

Adult male C57BL/6 mice (8–12 weeks) weighing 25–30 g were purchased from the Laboratory Animal Center of the Academy of Military Medical Sciences (Beijing, China) and housed under controlled-temperature and humidity conditions with a 12-h light/dark cycle. The animal protocol was approved by the Logistics University of PAP Ethics Committee for Animal Experimentation. All animal experimental procedures were executed in accordance with the rules and regulations of the Institutional Animal Care and Use Committee of the Logistics University of PAP and complied with the Guide for the Care and Use of Laboratory Animals. All efforts were made to minimize the animals suffering during the experimental procedures. Animal groups included normal, sham, MCAO, zVAD, GSK’872, and combination treatment (GSK’872 + zVAD) (*n* = 9 per group). The study design is described in Supplementary Figure S5.

### Middle cerebral artery occlusion (MCAO) model of focal ischemia

Focal cerebral ischemia was induced by transient occlusion of the middle cerebral artery (MCA) as previously described in mice^41^. Mice were anesthetized with isoflurane, and body temperature was maintained at 37 °C with an automatic warming blanket. Cerebral blood flow (CBF) was monitored by laser Doppler Flowmetry (LDF; Perimed Periflux 5010, Sweden). After making a neck midline incision, the right common carotid artery (CCA) and its bifurcation were exposed, then 1 6–0 poly-lysine coated monofilament nylon suture (Jialing Biotech, Guangzhou, China) was inserted into the internal carotid artery (ICA) along the CCA for approximately 1 cm to occlude MCA. CBF that dropped to >75% below the baseline was considered successfully occluded. After 1 h of occlusion, mice were re-anesthetized, and the suture was gently removed to reperfusion. Mice in the sham group underwent the same operation except for the suture insertion. MCAO mice were randomly assigned to different experimental groups.

### Drug administration

zVAD and GSK’872 were dissolved in DMSO (<0.1%) and diluted in saline. Drug concentrations were determined as previously described with some modifications^42, 43^. After the MCAO surgical procedure, mice in the MCAO, zVAD, GSK’872, and combination treatment groups were intraperitoneally (i.p.) injected with saline, zVAD (2.3 mmol/kg), GSK’872 (1.9 mmol/kg), and a combination of zVAD (2.3 mmol/kg) and GSK’872 (1.9 mmol/kg) at the onset of reperfusion, respectively. Drugs were administrated again at 24 and 48 h after reperfusion.

### Evaluation of neurological deficits

The modified neurological severity score (mNSS) was used to evaluate neurological functional deficits in mice (*n* = 9 per group) at 6, 24, 48, and 72 h after stroke. The mNSS is a composite of motor, sensory, balance and reflex tests with the maximum scores of 6, 2, 6, and 4 points, respectively. Thus, higher scores indicate more severe neurological dysfunction (13–18 = severe, 7–12 = moderate, and 1–6 = mild)^44^.

### Measurement of infarct volume

Mice were anesthetized and decapitated at 48 h post-stroke. Brains were removed and coronally cut into four 2-mm slices. Brain slices were subsequently incubated in 2% 2,3,5-triphenyltetrazolium chloride (TTC) solution (Sigma, St Louis, MO) at 37 °C for 30 min. After staining, slices were fixed with 4% of paraformaldehyde at 4 °C overnight. Infarct volume was then quantified and analyzed using computer software (Image Pro Plus 6.0, NIH, USA). The percentage of the infarct volume was calculated as follows: (infarct volume/the brain volume) × 100%.

### Immunofluorescence

Anesthetized mice were decapitated, and their brains were rapidly removed at 72 h after stroke. The brains were fixed with 4% paraformaldehyde for 24 h and dehydrated by 30% sucrose for 72 h at 4 °C. The brains were then cut into 5-μm coronal sections with a cryostat, and these sections were collected on coated glass slides. After air-drying, the sections were fixed with acetone for 15 min, permeabilized with PBS containing 0.5% Triton-X100 for 30 min, blocked by 2.5% goat serum for 30 min, and then incubated with anti-RIP1 rabbit primary antibody (1:100 dilution; Cell Signaling Technology, catalog #8737) overnight at 4 °C. Anti-rabbit secondary conjugated with CY3 (1:200 dilution; Life Technologies, catalog A12520) was subsequently added to slides and incubated for 1 h at 37 °C. After being washed with PBS, sections were incubated with FITC-tagged rabbit primary antibody against RIP3 (1:100 dilution; Abcam, catalog ab214227) overnight at 4 °C. Hoechst33342 (Dojindo Molecular Technologies, catalog JZ683) was used for nucleus visualization. Fluorescent images were captured using confocal microscopy (Leica TCS SP8, Germany).

### Western blotting assay

Mice were sacrificed, and their brains were rapidly removed at 24 h post-MCAO, whereas HT22 cells were collected at 9 h after OGD. For total protein extraction, brains and cells were lysed in 4% SDS buffer, homogenized on ice and stored at −80 °C. Protein concentrations were determined with a BCA protein assay kit (Cwbiotech, Beijing, China). Following this, protein was separated by 10% SDS-PAGE and transferred to nitrocellulose membrane. The membranes were blocked by 5% non-fat milk and incubated with primary antibodies against RIP1 (1:1000, Cell Signaling Technology, catalog #8737), RIP3 (1:1000, Abcam, catalog ab56164), MLKL (1:1000, Abcam, catalog ab194699), HIF-1α (1:1000, Abcam, catalog ab179483), and *β*-actin (1:5000, Proteintech, catalog 20536-1-AP) at 4 °C overnight. After being extensively washed with TBST, the membranes were incubated with anti-rabbit secondary antibodies (1:10000, KPL, catalog 074-1506) for 1 h at room temperature. Protein was developed with ECL reagent (GE Healthcare, Pittsburgh, USA) and visualized using an Amersham Imager 600 (GE Healthcare, Pittsburgh, USA). The density of bands was determined by Scion Image 4.0 software. *β*-actin was used as internal control.

### Statistical analysis

All data were presented as mean ± SD. Statistical analysis was performed using SPSS 13.0 software. We use *t*-test to compare statistical significance within two groups or one-way analysis of variance (ANOVA) followed by Tukey’s multiple comparison test for multiple groups comparison. *p* < 0.05 was considered to be statistically significant.

## Electronic supplementary material

Supplementary Video S1a

Supplementary Video S1b

Supplementary Video S2a

Supplementary Video S2b

Supplementary Video S3a

Supplementary Video S3b

Supplementary Video S3c

supplementary figure and video legends
