# Supplementary material for: Hypoxia-inducible factor-1 alpha is involved in RIP-induced necroptosis caused by in vitro and in vivo ischemic brain injury
Source: Sci Rep. 2017 Jul 19;7:5818. doi: 10.1038/s41598-017-06088-0 (PMC5517428; doi:10.1038/s41598-017-06088-0)
Supplement: Supplementary file 8 — supplementary figure and video legends [file 41598_2017_6088_MOESM8_ESM.pdf]

Hypoxia-inducible factor-1 alpha is involved in RIP-induced necroptosis caused by *in vitro* and *in vivo* ischemic brain injury

Xiao-Sa Yang [1]*, Tai-Long Yi [1]*, Sai Zhang [1]*, Zhong-Wei Xu [2], Ze-Qi Yu [1], Hong-Tao Sun [1], Cheng Yang [1], Yue Tu [1#], Shi-Xiang Cheng [1#]

[1] Tianjin Key Laboratory of Neurotrauma Repair, Institute of Traumatic Brain Injury and Neuroscience, Center for Neurology and Neurosurgery of Affiliated Hospital of the Logistics University of Chinese People's Armed Police Force (PAP), No. 220 ChengLin Road, HeDong District, Tianjin 300162, China

[2] Central Laboratory of Logistics University of PAP, No. 1 Huizhi Huan Road, DongLi District, Tianjin 300393, China

* These authors contributed equally to this work and should be considered co-first authors

[#] **Correspondence author**

Yue Tu

Tianjin Key Laboratory of Neurotrauma Repair, Institute of Traumatic Brain Injury and Neuroscience, Center for Neurology and Neurosurgery of Affiliated Hospital of the Logistics University of Chinese People's Armed Police Force (PAP), No. 220 ChengLin Road, HeDong District, Tianjin 300162, China

Tel: +86-22-60577173

Fax: +86-22-24715698

E-mail: ytumail@vip.126.com

Shi-Xiang Cheng

Tianjin Key Laboratory of Neurotrauma Repair, Institute of Traumatic Brain Injury and Neuroscience, Center for Neurology and Neurosurgery of Affiliated Hospital of the Logistics University of Chinese People’s Armed Police Force (PAP), No. 220 ChengLin Road, HeDong District, Tianjin 300162, China

Tel: +86-22-60577171

Fax: +86-22-60577175

E-mail: shixiangcheng@vip.126.com

Supplementary Figure and video legends

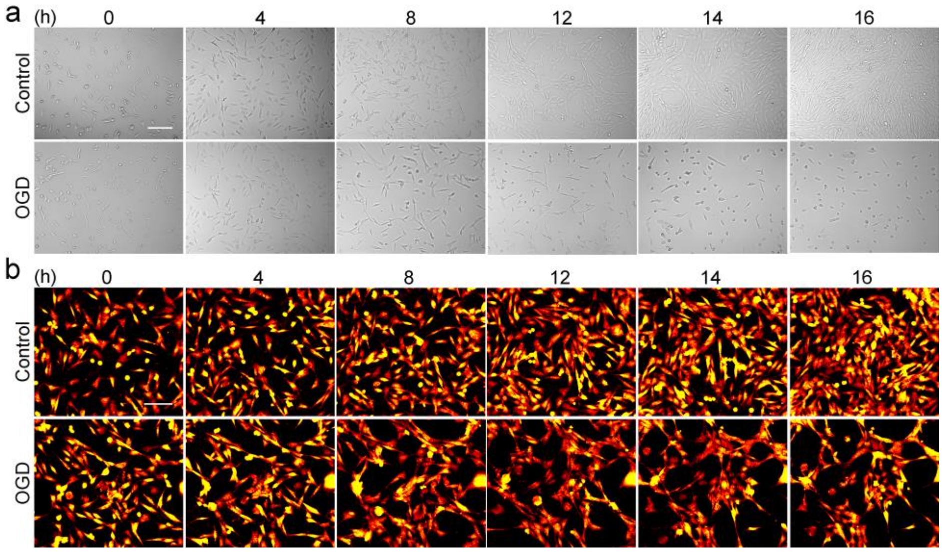

Supplementary Figure S1. HT-22 cell morphology changes during OGD. Images of HT-22 cells were captured by light microscope (**a**) and digital

hologram microscope (**b**) at 0, 4, 8, 12, 14, 16 h of OGD. Scale bars = 100 μm.

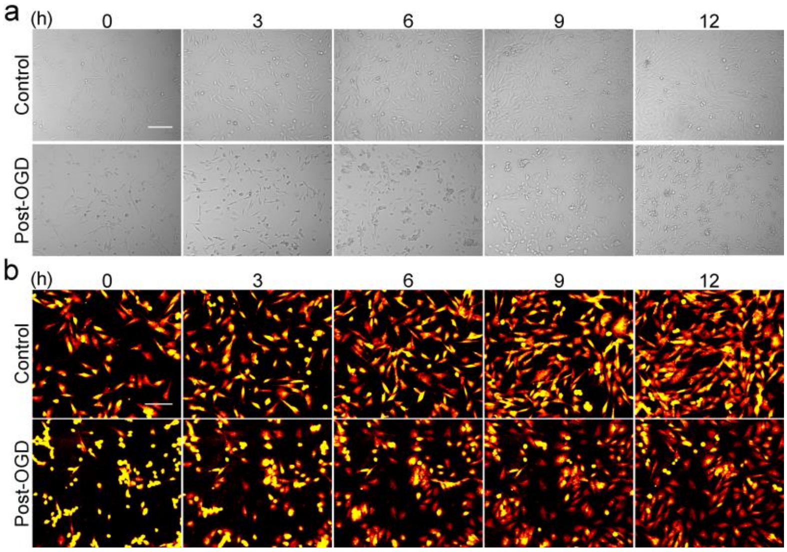

Supplementary Figure S2. HT-22 cell morphology changes during reoxygenation. Light microscope (**a**) and digital hologram microscope (**b**) were used to obtain images of HT-22 cells at 0, 3, 6, 9, and 12 h of reoxygenation. Scale bars = 100 μm.

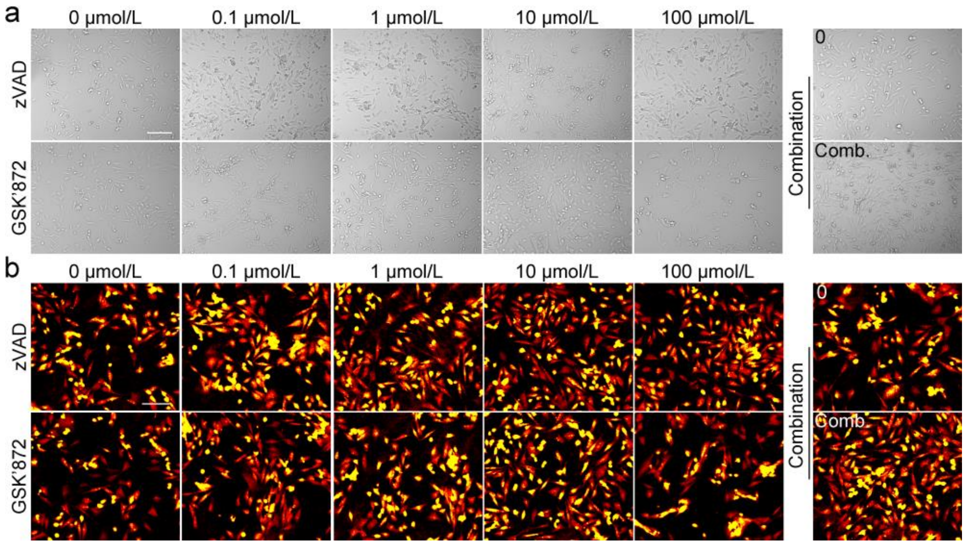

Supplementary Figure S3. The morphologic alterations of HT-22 cells with different treatment during oxygenation. HT-22 cells were treated with

different concentrations of zVAD, GSK’872, and combination of 10 μM zVAD and GSK’872 during 9 h of reoxygenation and the morphologic changes were detected by light microscope (**a**) and digital hologram microscope (**b**). Scale bars = 100 μm.

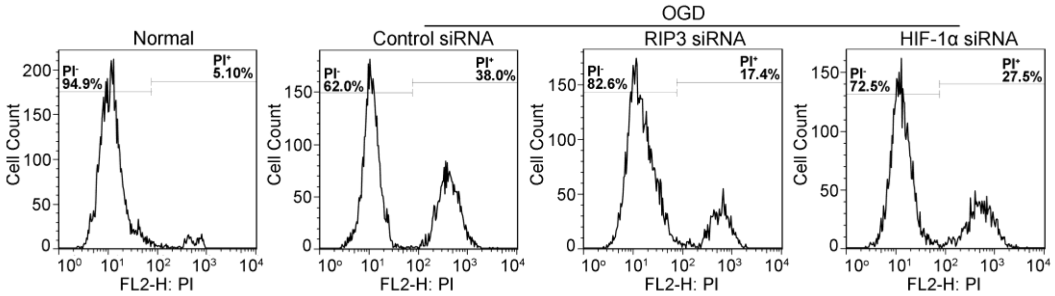

Supplementary Figure S4. The representative flow cytometry images of HT-22 cells with different siRNA transfection. HT-22 cells were transfected with different siRNA for 24 h before OGD modeling. The dead cells were stained with PI and detected by flow cytometry.

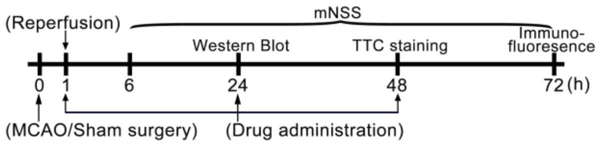

Supplementary Figure S5. The mice experiment schedule. Focal cerebral ischemia was induced by MCAO and reperfusion was initiated after 1 h of occlusion. Drug administration was carried out at the beginning of reperfusion and repeated at 24 and 48 h after reperfusion. Neurological deficits were evaluated by mNSS for 72 h. Mice were sacrificed at 24, 48, and 72 h after MCAO to detect the relative expression of proteins by Western Blot, infarct volume by TTC staining and protein co-localization by immunofluorescence.

Supplementary Video S1. Digital hologram microscopy was used to acquire dynamic videos showing normal cell growth (**a**) and changes in HT-22 cells exposed to OGD for 16 h (**b**).

Supplementary Video S2. Digital hologram microscopy was used to acquire videos showing normal cell growth (**a**) and changes in HT-22 cells during post-OGD reoxygenation for 12 h (**b**).

Supplementary Video S3. Digital hologram microscope was used to record (at 20 min intervals) the dynamic changes of HT-22 cells treated with 10 μM zVAD (**a**), 10 μM GSK'872 (**b**), and a combination of 10 μM zVAD and GSK'872 (**c**) during 9 h of reoxygenation.
